# Biphasic Modulation of NMDA Receptor Function by Metabotropic Glutamate Receptors

**DOI:** 10.1523/JNEUROSCI.1000-18.2018

**Published:** 2018-11-14

**Authors:** Nathanael O'Neill, Catherine McLaughlin, Noboru Komiyama, Sergiy Sylantyev

**Affiliations:** ^1^Centre for Clinical Brain Sciences, University of Edinburgh, EH16 4SB, Edinburgh, United Kingdom,; ^2^Centre for Neuroregeneration, University of Edinburgh, EH16 4SB, Edinburgh, United Kingdom, and; ^3^Department for Clinical and Experimental Epilepsy, Institute of Neurology, University College London, London WC1N 3BG, United Kingdom

**Keywords:** cerebellar granule cells, dentate gyrus granule cells, Homer protein, inter-receptor crosstalk, metabotropic glutamate receptors, NMDA receptors

## Abstract

A recently reported rapid potentiation of NMDA receptors by Group I metabotropic glutamate receptors (mGluRIs) via a Homer protein link is distinct from the classical, relatively slow inhibitory G-protein-associated signaling triggered by mGluRI activation. The relationship between these two mechanisms remains unknown. Here, we focused on the mGluRI-dependent modulation of NMDAR response in hippocampal dentate gyrus granule cells and cerebellar granule cells of C57BL6-J mice and found that these two contrasting mechanisms overlap competitively on the time scale from hundreds of milliseconds to seconds, with the net effect depending on the cell type. At a shorter time interval (units of millisecond), the Homer-mediated signal from mGluRIs prevails, causing upregulation of NMDAR function, in both dentate gyrus granule cells and cerebellar granule cells. Our results shed light on the possible mechanisms of anti-schizophrenia drugs that disrupt Homer-containing protein link.

**SIGNIFICANCE STATEMENT** Here we study modulation of NMDA receptors triggered by activation of metabotropic glutamate receptors Group I via two distinct pathways: classical G-protein signaling system and newly discovered high-speed modulatory mechanism associated with Homer-protein-containing direct molecular link. We found that these two contrasting mechanisms overlap competitively on the time scale from hundreds of milliseconds to seconds, with the net effect depending on the cell type. We have also found that both crosstalk mechanisms cause significant changes in synaptic strength and plasticity. Our results resolve an apparent discrepancy between earlier studies that demonstrated contradictive effects of Homer-containing protein link disruption on NMDA receptor signaling. On top of that, our data provide a plausible explanation for unclear action mechanisms of anti-schizophrenia drugs.

## Introduction

Neuronal metabotropic glutamate receptors Group I (mGluRIs) are classically viewed as the modulators (predominantly augmentative) of NMDA receptors (NMDARs); G-protein signaling cascades are the transfer mechanism of this modulation (Rossi et al., 1996; Kunishima et al., 2000; Pin and Acher, 2002). This signaling mechanism can be interrupted by blocking various stages of the G-protein cascade. Pertussis toxin (PeTX) acts as a blocker of G_i_, G_o,_ and G_t_ proteins (Kost et al., 1999), enabling research of the mGluRI-NMDAR interaction cleared from G-protein signaling effects.

The second, more recently discovered, mechanism of mGluRI-NMDAR crosstalk is signal transfer via the Homer proteins. Homer protein family contains a C-terminal coiled-coil dimerization domain (“long” Homers), except for Homer1a and Ania-3 (“short” Homers), which lack this domain and, as a result, are unable to perform a scaffolding role linking other proteins. Homer proteins bind to the mGluRIs (Brakeman et al., 1997), and to the proteins of the Shank family (Tu et al., 1999). The Shank proteins, in turn, connect to NMDARs via the protein PSD-95, establishing a direct molecular link between mGluRIs and NMDARs (Bertaso et al., 2010). Overexpressed Homer1a disrupts mGluRI-NMDAR protein connection by outcompeting “long” Homers on the binding site of mGluRIs, thus providing an experimental approach where the mGluRI-NMDAR modulation signal can pass through cytoplasmic biochemical cascades only.

We have previously shown that, at a short time scale (low milliseconds), mGluRI activation in cerebellar granule cells (CGCs) potentiates NMDARs in intact cells but has no effect when the Homer molecular interlink has been disrupted by Homer1a overexpression (Sylantyev et al., 2013). An earlier study, however, showed that at longer intervals (hundreds of milliseconds to seconds), the activation of mGluRIs in CGCs does not affect NMDARs when the Homer scaffold is intact, but depresses NMDAR responses when the Homer-containing interlink is abolished, that is, mGluRI-NMDAR modulatory signal is delivered exclusively through G-protein cascades (Bertaso et al., 2010). In contrast, long-term NMDAR effects were repeatedly shown to be potentiated by mGluRIs in experimental cell systems (*Xenopus* oocytes) (Skeberdis et al., 2001) and in brain areas other than the cerebellum, such as the subthalamic nucleus (Awad et al., 2000) and hippocampus (Harvey and Collingridge, 1993; Fitzjohn et al., 1996; Naie and Manahan-Vaughan, 2004).

It has been widely accepted that NMDAR hypofunction is one of the key factors provoking schizophrenia development (Steullet et al., 2016; Nakazawa et al., 2017). Another important element, in which dysfunction is tightly associated with the propagation of schizophrenia, are mGluRIs (Cleva and Olive, 2011). In particular, the inhibition of metabolic signaling delivered by G-proteins has been implicated in propagating schizophrenia symptoms (Chowdari et al., 2002; Williams et al., 2004). In turn, Homer1-knock-out animals, in which a direct mGluRI-NMDAR link is absent, exhibit a wide spectrum of abnormalities that are consistent with schizophrenia symptoms (Szumlinski et al., 2005), thus suggesting Homer as an actor preventing schizophrenia development. However, anti-schizophrenia drugs, such as haloperidol and clozapine, upregulate the synthesis of Homer1a protein, which destroys the Homer-containing mGluRI-NMDAR link (Polese et al., 2002).

Here, we aimed to resolve these apparent functional discrepancies and to clarify the interaction of overlapping signals delivered through (1) rapid Homer-mediated and (2) slower G-protein-mediated pathways of mGluRI-NMDAR crosstalk. We hypothesized that mGluRIs, when activated, could modulate NMDARs simultaneously via the two pathways, Homer- and G-protein-controlled. The pathway that prevails is dependent on the time scale of activation: the fast Homer-transduced effect is more prominent at short-term intervals after mGluRI activation, whereas the slower, G-protein-delivered effect has an advantage on long-term intervals. To test the hypothesis, we set out to investigate the mGluRI-NMDAR interaction in two distinct cell types, where earlier studies demonstrated different long-term effects of mGluRIs on NMDAR response: potentiation (in hippocampal dentate gyrus granule cells, DGCs) (Naie and Manahan-Vaughan, 2004) and suppression (in CGCs) (Bertaso et al., 2010).

## Materials and Methods

### 

#### Generation of cell cultures

##### Obtaining cells.

Cultures were generated from E17.5, C57BL6-J mice. Pregnant mother was killed with cervical dislocation according to UK Animals (Scientific Procedures) Act 1986 Schedule 1. Embryos were removed by caesarean section and decapitated in Hanks Balanced Salt Solution (Invitrogen, 14170–88) containing 5% penicillin-streptomycin (Invitrogen, 15070–063) on ice. Skulls were removed and brain tissue dissected under a light microscope where the cerebellum were separated followed by hippocampi removal from the embryo forebrain. Tissue was then exposed to enzymatic digestion using Papain (Worthington, LK003176) following mechanical dissociation in complete DMEM (Invitrogen, 11960–044) containing 10% FBS (Invitrogen, 10500) and 5% penicillin-streptomycin. Tissue was washed with DMEM and pelleted twice before being resuspended in complete Neurobasal media (Invitrogen, 21103049) containing 10% B27 (Invitrogen, 17504), 5% PenStrep and 0.25% L-gluatamine (Invitrogen, 25030). Cells were plated at a density of 2–5 × 10^4^ cells/ml on glass coverslips that had been coated with poly-D-lysine (Sigma, P7280) and Laminin protein (Invitrogen, 2301015). Cultures were maintained in a humidified incubator at 37°C, <5% CO_2_.

##### Transfection.

Granule cells were transfected at *DIV7* with pRK5-Homer1a overexpression vector and mCherry fluorescent marker vector (Clontech, 632523) using Lipofectamine 2000 (Thermo Fisher Scientific, 11668027); 250 ng DNA per vector was added to Optimem and then incubated with Lipofectamine at room temperature before being dripped onto the cultures and left to recover for 5 d before recordings were taken.

#### Electrophysiology

Visualized patch-clamp recordings from cultured granule cells were performed using an infrared differential interference contrast imaging system. The perfusion solution contained the following (in mm): 119 NaCl, 2.5 KCl, 1.3 Na_2_SO_4_, 2.5 CaCl_2_, 26.2 NaHCO_3_, 1 NaH_2_PO_4_, 22 glucose and was continuously gassed with 95% O_2_/5% CO_2_, pH 7.35 (290–298 mOsm). The intracellular pipette solution for voltage-clamp experiments contained the following (in mm): 120.5 CsCl, 10 KOH-HEPES, 2 EGTA, 8 NaCl, 5 QX-314 Br^−^ salt, 2 Na-ATP, 0.3 Na-GTP. For current-clamp recordings, intracellular pipette solution contained the following (in mm): 126 K-gluconate, 4 NaCl, 5 HEPES, 15 glucose, 1 K_2_SO_4_ · 7H_2_O, 2 BAPTA, 3 Na-ATP. pH was adjusted to 7.2 and osmolarity adjusted to 295 mOsm.

In experiments on DGCs, we selected for patching neurons, which morphology reproducing that reported for mature DGCs in culture: cells with roundish body of 10–15 μm and bipolar neurites (Lowenstein and Arsenault, 1996). Each patched cell was first tested for electrophysiological properties. Further experimental procedures were performed at cells reproducing all three characteristics of mature DGCs: whole-cell capacitance of 15–25 pF, membrane potential of −65 to 75 mV, and input resistance of 150–300 mΩ (Spampanato et al., 2012).

In experiments on CGCs, we selected for patching cells with mature morphology reproducing that observed in our earlier study: small (∼5 μm) neurons with short dendrites (Sylantyev et al., 2013), and then monitored their electrophysiological properties. We continued experimental protocol on cells reproducing all three of the following characteristics: whole-cell capacitance of 2–5 pF, membrane potential −60 or 70 mV, input resistance 5–8 GΩ (Hevers and Lüddens, 2002; Sylantyev et al., 2013).

##### Outside-out and nucleated patch recordings.

Outside-out patches and cell membrane bags containing intact nucleus and cytoplasm (nucleated patches [NPs]) were pulled from DGCs and CGCs, and recordings were performed in voltage-clamp mode (V_hold_ − 70 mV). Solution exchange experiments were performed as described in our previously published protocol (Sylantyev and Rusakov, 2013). Briefly, we used a θ-glass application pipette with ∼200-μm-tip diameter attached to the micromanipulator. The position of the pipette was controlled by piezoelectric element (the speed of switch was 50–100 μs). One pipette channel was filled with the bath aCSF solution; another channel had glutamate receptor ligands. Pressure was regulated by a PDES-02DX pneumatic micro ejector (npi) using compressed nitrogen separately in each of two channels. Solutions with NMDA + glycine, NMDA + glycine + DHPG, and NMDA + glycine + FTIDC + Fenobam were exchanged in a pipette channel (7–12 s) during the exposure of nucleated patch to the bath solution channel.

##### Whole-cell recordings.

Whole-cell EPSPs were measured in current-clamp mode in the presence of the following set of ligands: picrotoxin (50 μm), NBQX (20 μm), strychnine (1 μm), and CGP-55845 (1 μm). Recordings were performed at 32°C–34°C; the patch pipette resistance was 3–7 mΩ, depending on particular experimental conditions. Series resistance was monitored throughout experiments using a 5 mV step command; cells with unstable series resistance (>25 mΩ) or unstable holding current were rejected.

To assess modulation of synaptic efficacy in polysynaptic signaling pathways, we used an experimental approach tested on hippocampal cell cultures earlier (Bi and Poo, 1999). Briefly, evoked EPSCs were recorded from the neuron in a network of 20–30 cells (cut from surrounding cell culture by blunt electrode) after current injection applied to another patched neuron nearby. For the sake of clarity, we isolated segments of neuronal networks containing only one Homer1a-overexpressing cell, which was recorded when disruption of Homer interlink was studied; or no Homer1a-overexpressing cells when we studied G-protein-delivered crosstalk. Perfusion solution in this experiment did not contain receptor antagonists. Each EPSC component propagated by recorded neuron was interpreted as a signal delivered through separate polysynaptic pathway with a specific transmission delay. To quantify the impact of mGluRI-NMDAR crosstalk on synaptic efficacy, we measured probability of EPSC component occurrence (*P*) in control and after series of paired stimuli, when G-protein signaling cascade and/or Homer protein interlink were interrupted. To allow registration of both increase and decrease of *P*, in the beginning of the experiment, stimulation was adjusted to generate *P* in an interval 25% < *P* < 75%. If under control conditions EPSC component had *P* out of this interval, the component was not used in further statistical calculations.

#### Fluorescent imaging and immunostaining

DIV11–14 mCherry-positive cells were selected for tests of Homer1a-overexpression effects using induced fluorescence (λ_em_ 580 nm). An experimental test of calbindin-D_28k_ presence was done with a method described previously (Müller et al., 2005). To do this, whole-cell patch clamp was performed with the GFP added to intracellular solution. After patching and recording of electrophysiological parameters (membrane potential, membrane capacitance, input resistance), patching pipette was withdrawn and a fluorescent image of the patched cell taken (λ_em_ 510 nm). Hereupon coverslips were placed to 4% PFA for 12 h at room temperature and then incubated at 4°C for 24 h with monoclonal mouse anti-calbindin-D_28k_ antibodies (1:1000) in TBS with 0.3% Triton X-100. Next, biotin-conjugated sheep anti-mouse antibodies (1:200) and streptavidin-conjugated Cy5 (1:300) were applied sequentially overnight; then Cy5 fluorescence imaged (λ_em_ 670 nm).

#### Acquisition and analysis

Recordings were obtained using a MultiClamp 700B amplifier (Molecular Devices), filtered at 4–8 kHz, digitized at 10 kHz, and stored on a PC. pClamp/Clampfit 10x software (Molecular Devices) was used for data storage and off-line analysis.

##### Analysis of the macroscopic currents.

Activation of NMDARs with high concentrations of NMDA and glycine (50 μm each) at outside-out and nucleated patches evoked macroscopic responses, where “peak response” was obtained as a difference between baseline (average for 50 ms before application of NMDAR ligands) and maximum evoked current; “stable” or “equilibrated” response was obtained as a difference between baseline and stable current generated at 200–800 ms interval ∼4 s after start of continuous application of NMDAR ligands.

##### Analysis of the single-channel recordings.

Activation of NMDARs with low concentration of NMDA and glycine (1 μm each) at outside-out and nucleated patches evoked single-channel openings to three conductance levels: 51.9 ± 7.8 pS, 37.2 ± 6.4 pS, and 22.1 ± 8.3 pS. The larger conductance level contributed 91.9% of the single-channel current, whereas medium and low subconductance levels contributed 6.2% and 1.9% of the current, respectively; thus, the larger main conductance level was used when (possible) changes of single-channel conductance were compared under different experimental conditions. Because it was virtually impossible to determine accurately the number of channels in a nucleated patch, the open probability was obtained as the total open probability of N channels (NP_o_), calculated as the proportion of the channel total open time to the duration of recording. Because NP_o_ in individual patches widely varied (from 0.02 to 0.2), for statistical calculations we used values normalized to control (NP_o_ generated by NMDA+Gly in corresponding patch). With the low (1 μm) NMDA and glycine concentrations used in this study, the majority of channel events were single-level events. In a case where there were multiple levels of channel openings, only levels with the highest conductance was analyzed. This prevented us from overestimation of NP_o_ increase because in multichannel patch increased NP_o_ would be accompanied by increase of proportion of multilevel events. To inspect the stationarity of channel activities, conductance, NP_o_, and the open time were followed in control experiments for up to 15 min; no time-dependent alterations were detected.

Strychnine, FTIDC, Fenobam, GGP-55845, NBQX, APV, and PeTX were purchased from Tocris Bioscience. All other chemicals, biotin-conjugated sheep anti-mouse antibodies, and monoclonal anti-calbindin-D_28k_ mouse antibodies were purchased form Sigma-Aldrich. Streptavidin-conjugated Cy5 and GFP were purchased from Thermo Fisher Scientific.

All data are given as mean ± SEM. Statistical comparisons were made with Student's unpaired *t* test, unless difference is indicated in the text; *p* ≤ 0.05 was taken as a threshold of significance.

Figures 9*E* and 10 were created with elements from PPT Neuroscience Toolkit 2010 (Motifolio).

## Results

### mGluRIs modulate equilibrated NMDAR response

First, we tested the mGluRI-NMDAR interaction on nucleated membrane patches where solutions containing mGluRI and NMDAR ligands were exchanged on the same patch, with registration of stable response amplitude. Three combinations of ligands were applied at the same patch sequentially: first, 50 μm NMDA + 50 μm glycine (Gly) to activate only NMDARs; second, 50 μm NMDA + 50 μm Gly + 50 μm DHPG to activate NMDARs and mGluRIs; being compared with response generated by NMDARs only, this should reveal mGluRIs modulatory effect on NMDARs; third, 50 μm NMDA + 50 μm Gly + inverse agonists of mGluRIs: 100 nm FTIDC and 1 μm Fenobam (FB); comparison to “NMDARs only” response should reveal whether mGluRIs modulate NMDARs due to spontaneous activation. Each solution was applied for 4 s, as this time was enough to stabilize NMDAR response amplitude. This protocol was repeated on intact cells and cells overexpressing Homer1a with standard intracellular solution and intracellular solution containing PeTX (1 μg/ml), which allowed us to register four modes of mGluRI-NMDAR crosstalk: mode i, intact cell, standard internal solution: both Homer- and G-protein signaling pathways are active; mode ii, intact cell, internal solution with PeTX: only Homer signaling pathway is active; mode iii, Homer1a-overexpressing cell, standard internal solution: only G-protein signaling pathway is active; mode iv, Homer1a-overexpressing cell, internal solution with PeTX: both signaling pathways are blocked. To account for individual patch characteristics, for statistical calculations, the response amplitudes were normalized to the amplitude of response generated by the application of 50 μm NMDA + 50 μm Gly.

**Figure 1.**
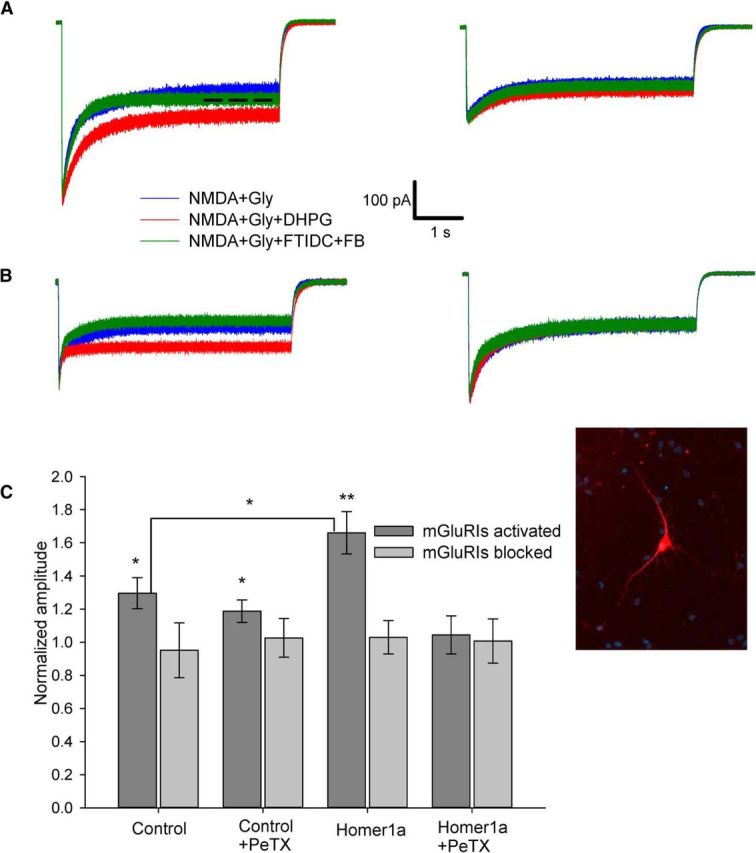
mGluRIs potentiate amplitude of equilibrated NMDAR response through G-protein and Homer signaling pathways in DGCs. ***A***, Nucleated patches excised from control cell. Left, Standard intracellular solution (both signaling pathways are active). Right, PeTX added to intracellular solution (only Homer pathway active). ***B***, Nucleated patch excised from the Homer1a-overexpressing cell. Left, Standard intracellular solution (only G-protein signaling pathway active). Right, PeTX added to intracellular solution (both signaling pathways are blocked). Color codes of applied ligand cocktails and scale bars apply to ***A*** and ***B***. Dashed line indicates time interval where response amplitude was calculated. ***C***, Statistical summary of ***A*** and ***B***. Response amplitudes are normalized to amplitude generated by NMDA+Gly. Significance of difference from unity: *p < 0.05; **p < 0.01; Student's *t* test. Inset, Fluorescent image of cultured DGC cotransfected with Homer1a and mCherry.

Nucleated patches from DGCs, subjected to this experimental protocol, demonstrated a significant impact of mGluRIs on NMDAR function delivered through both Homer- and G-protein signaling chains. In contrast, the application of mGluRI inverse agonists, as in all further experiments, did not cause any significant modulation of effect generated by NMDA+Gly (Fig. 1). Normalized response amplitude: mode i, 1.29 ± 0.094 when mGluRIs were activated by DHPG versus 0.95 ± 0.155 when mGluRIs were blocked by FTIDC+FB; mode ii, 1.19 ± 0.068 versus 1.03 ± 0.12; mode iii, 1.66 ± 0.13 versus 1.02 ± 0.1; mode iv, 1.04 ± 0.11 versus 1.01 ± 0.13. Significance of difference from unity, when mGluRIs were activated: mode i, *p* = 0.036, *n* = 9; mode ii, *p* = 0.034, *n* = 6; mode iii, *p* = 0.004 (*n* = 6, paired Student's *t* test). The normalized mGluRI-modulated response in mode iii was also significantly higher than that of mode i: 1.66 ± 0.13 versus 1.3 ± 0.094, *p* = 0.044 (*n* = 6, Student's *t* test; Fig. 1*C*).

**Figure 2.**
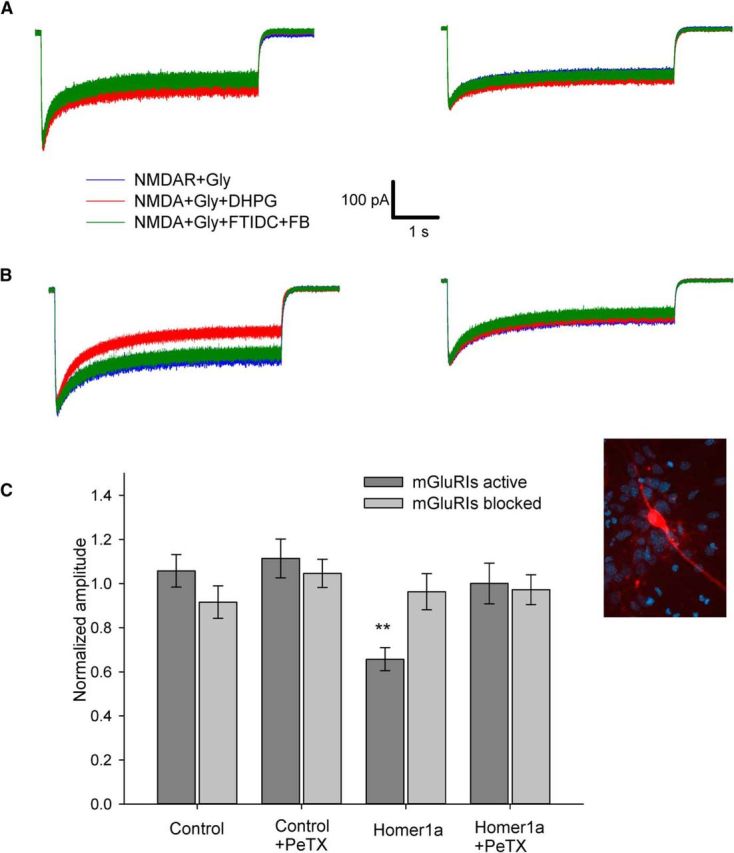
mGluRIs downregulate amplitude of equilibrated NMDAR response through G-protein signaling pathway in CGCs. ***A***, Nucleated patch excised from control cell. Left, Standard intracellular solution. Right, PeTX added to intracellular solution. ***B***, Nucleated patch excised from the Homer1a-overexpressing cell. Left, Standard intracellular solution. Right, PeTX added to intracellular solution. Color codes of applied ligand cocktails and scale bars apply to ***A*** and ***B***. ***C***, Statistical summary of ***A*** and ***B***. Response amplitudes are normalized to amplitude generated by NMDA+Gly. Significance of difference from unity: **p < 0.01; paired Student's *t* test. Inset, Fluorescent image of cultured CGC cotransfected with Homer1a and mCherry.

When a similar experiment was repeated on nucleated patches from CGCs (Fig. 2), only G-protein signaling pathway, being activated alone (mode iii), generated a significant modulatory (downregulatory) effect. Normalized response amplitude: mode i, 1.06 ± 0.07 when mGluRIs were activated versus 0.92 ± 0.07 when mGluRIs were blocked; mode ii, 1.11 ± 0.08 versus 1.05 ± 0.06; mode iii, 0.66 ± 0.05 versus 0.96 ± 0.08; mode iv, 1.00 ± 0.09 versus 0.97 ± 0.07. Significance of difference from unity when mGluRIs were activated for mode iii: *p* = 0.016 (*n* = 6, paired Student's *t* test); in all other cases, no significant difference was observed (Fig. 2*C*).

**Figure 3.**
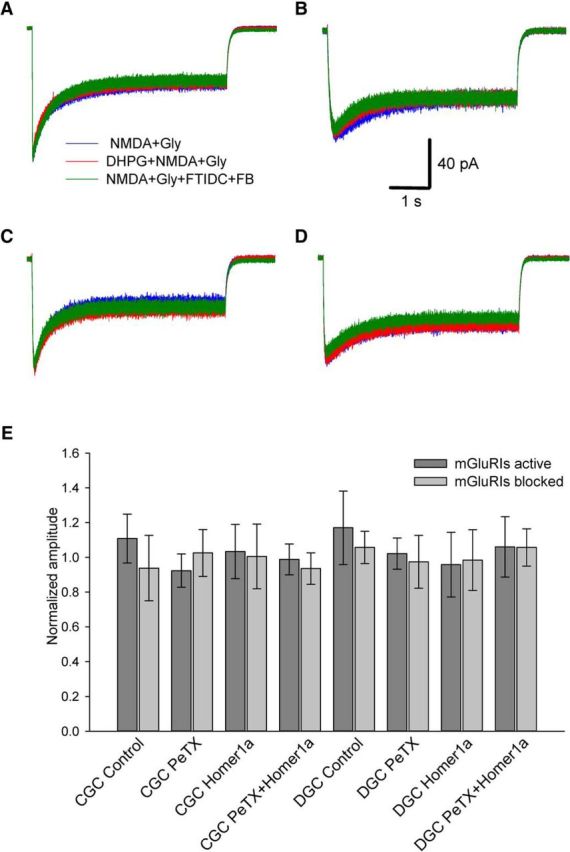
mGluRI-NMDAR crosstalk is absent in outside-out patches. ***A–D***, Example traces from experimental protocols where significant mGluRI effect was observed in nucleated patches (see Figs. 1*C*, 2*C*). ***A***, Outside-out patch from intact DGC, standard intracellular solution. ***B***, Outside-out patch from intact DGC, internal solution with PeTX. ***C***, Outside-out patch from DGC overexpressing Homer1a, standard internal solution. ***D***, Outside-out patch from intact CGC, internal solution with PeTX. Scale bars and color codes apply to ***A–D***. ***E***, Statistical summary on normalized response amplitudes for i, ii, iii, and iv experimental modes in DGCs and CGCs. Amplitudes normalized to the value generated by NMDA+Gly.

As a control, we tested this experimental protocol on outside-out patches excised from DGCs and CGCs, where both Homer- and G-protein signaling chains were supposed to be destroyed. As expected, in this experiment, any kind of pharmacological manipulations did not exert any significant effect (Fig. 3).

### mGluRIs modulate single-channel opening probability of NMDARs

**Figure 4.**
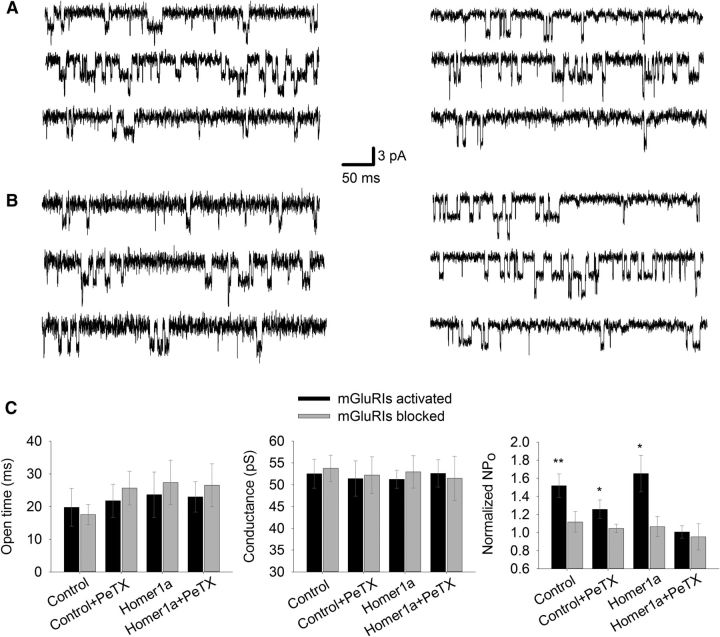
mGluRIs modulate NMDAR opening probability in DGCs. ***A***, Control cell. ***B***, Homer1a-overexpressing cell. ***A***, ***B***, Left, Standard intracellular solution. Right, PeTX added to intracellular solution. Top to bottom, NMDA+Gly, NMDA+Gly+DHPG, NMDA+Gly+FTIDC+FB. Scale bars apply to ***A*** and ***B***. ***C***, Statistical summary on single-channel functional parameters for ***A*** and ***B***. Significance of difference from unity: *p < 0.05; **p < 0.01; Student's *t* test.

After studying mGluRIs' impact on macroscopic NMDAR response, we asked how mGluRIs modulate the functional characteristics of individual NMDAR ion channels. To clarify this, we repeated the same experimental protocol, but with lowered concentrations of NMDA (1 μm) and Gly (1 μm) to make single-channel openings visible. Channel conductance, average open time, and opening probability were used as quantitative characteristics of the effect of mGluRIs (Fig. 4).

In DGCs, the average open time (13.7 ± 2.6 ms) and single-channel conductance (52.1 ± 2.4 pS) were indistinguishable between recordings in modes i–iv (Fig. 4*C*). However, data on opening probability demonstrated a significant upregulation of NMDAR function in i–iii modes, when normalized to control: mode i, 1.52 ± 0.13 when mGluRIs were activated versus 1.12 ± 0.11 when mGluRIs were blocked; mode ii, 1.26 ± 0.1 versus 1.03 ± 0.03; mode iii, 1.65 ± 0.2 versus 1.06 ± 0.11; mode iv, 1.01 ± 0.07 versus 0.95 ± 0.15. Significance of difference from unity when mGluRIs were activated for modes i–iii: *p* = 0.002, *p* = 0.029, and *p* = 0.013, respectively (*n* = 6 for all cases, Student's *t* test). In mode iv, no significant difference from unity was observed (Fig. 4*C*).

**Figure 5.**
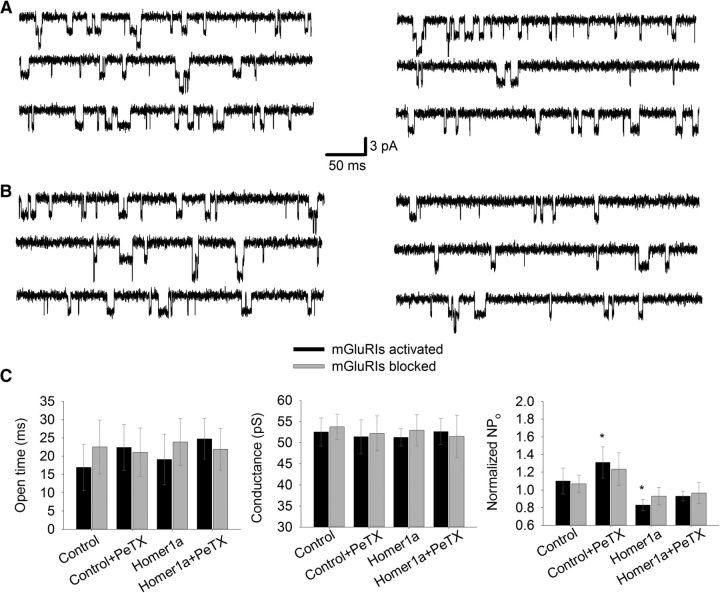
mGluRIs modulate NMDAR opening probability in CGCs. ***A***, Control cell. ***B***, Homer1a-overexpressing cell. ***A***, ***B***, Left, Standard intracellular solution. Right, PeTX added to intracellular solution. Top to bottom: NMDA+Gly, NMDA+Gly+DHPG, NMDA+Gly+FTIDC+FB. Scale bars apply to ***A*** and ***B***. ***C***, Statistical summary on single-channel functional parameters for ***A*** and ***B***. Significance of difference from unity: *p < 0.05; Student's *t* test.

In CGCs, similar to DGCs, the disruption of G-protein- and Homer-containing mGluRI-NMDAR crosstalk did not result in significant changes of average open time (11.9 ± 3.2 ms) and single-channel conductance (51.6 ± 3.7 pS) (Fig. 5). However, in CGCs, experimental modes ii and iii showed a significant mGluRIs effect on opening probability (Fig. 5*C*): mode i, 1.1 ± 0.15 when mGluRIs were activated versus 1.07 ± 0.1 when mGluRIs were blocked; mode ii, 1.31 ± 0.18 versus 1.23 ± 0.19; mode iii, 0.83 ± 0.06 versus 0.93 ± 0.1; mode iv, 0.93 ± 0.06 versus 0.96 ± 0.12. Significance of difference from unity when mGluRIs were activated for modes ii and iii: *p* = 0.042 and *p* = 0.037, respectively (*n* = 6 for both cases, Student's *t* test).

### Rapid potentiation of NMDAR effect is delivered exclusively through Homer-containing interlink

After experiments on long intervals, where Homer- and G-protein-delivered effects overlapped, we tested mGluRI-NMDAR modulation at short intervals (units of milliseconds). We presumed that, under these conditions, the G-protein-delivered effects might not have enough time to fully develop, as was observed previously (Sylantyev et al., 2013).

**Figure 6.**
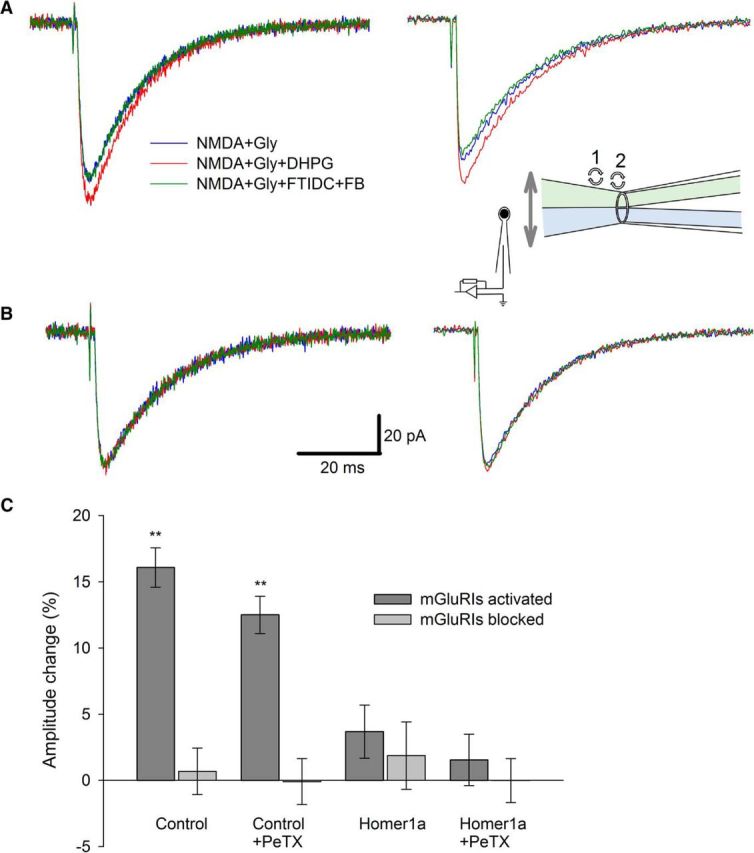
mGluRIs potentiate rapid NMDAR response through Homer signaling pathway only; nucleated patches excised from DGCs. ***A***, Control cell. Left, Standard intracellular solution. Right, PeTX added to intracellular solution. ***B***, Homer1a-overexpressing cell. Left, Standard intracellular solution. Right, PeTX added to intracellular solution. Color codes of applied ligand cocktails and scale bars apply to ***A*** and ***B***. Each trace is an average of 3–5. Inset, Illustration of rapid solution application system (schematic) with θ-glass pipette, which applies two different solutions at nucleated patch placed at a patch pipette. Numbers denote sequence of drug mixture replacements in θ-glass pipette channels: 1, NMDA+Gly → NMDA+Gly+DHPG; 2, NMDA+Gly+DHPG → NMDA+Gly+FTIDC+FB. During solution replacement time periods, patch was exposed to solution flowing from “bottom” channel. ***C***, Statistical summary on response amplitudes in ***A*** and ***B***. Significance of difference from zero: **p < 0.01; Student's *t* test.

**Figure 7.**
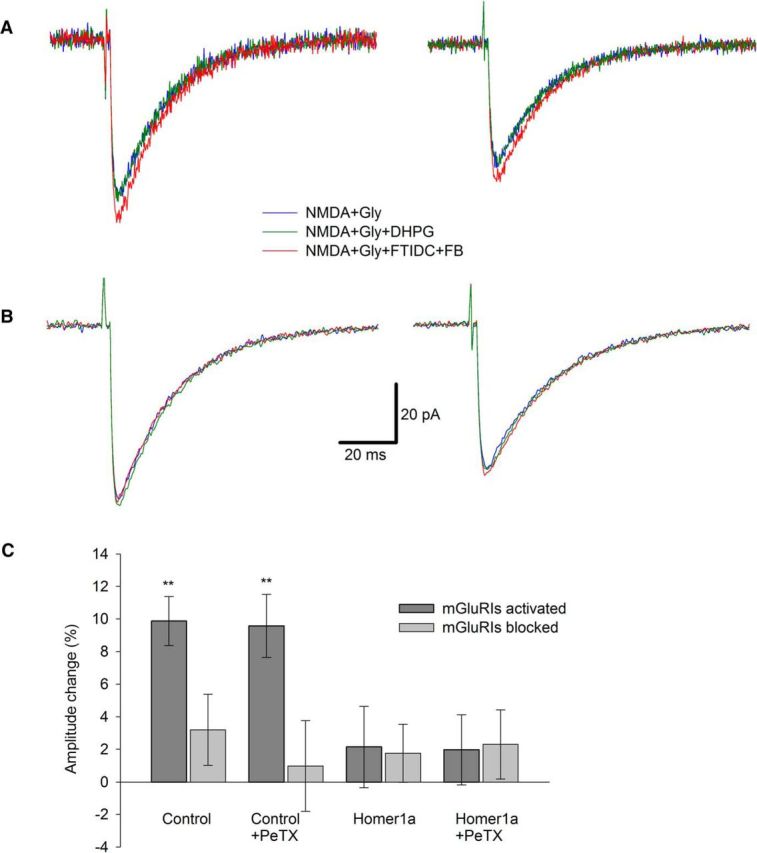
mGluRIs potentiate rapid NMDAR response through Homer signaling pathway only; nucleated patches excised from CGCs. ***A***, Control cell. Left, Standard intracellular solution. Right, PeTX added to intracellular solution. ***B***, Homer1a-overexpressing cell. Left, Standard intracellular solution. Right, PeTX added to intracellular solution. Color codes of applied ligand cocktails and scale bars apply to ***A*** and ***B***. Each trace is an average of 3–5. ***C***, Statistical summary on response amplitudes in ***A*** and ***B***. Significance of difference from zero: **p < 0.01; Student's *t* test.

We found in both DGCs and CGCs, after rapid (∼1 ms) application of agonists, that the activation of mGluRIs has increased peak amplitudes of NMDAR response independently of PeTX presence in the internal solution. For DGCs (Fig. 6): mode i, DHPG in applied solution increased the response amplitude by 16.09 ± 1.48% versus 0.68 ± 1.75% when the applied solution contained FTIDC+FB; mode ii, 12.52 ± 1.42% versus −0.1 ± 1.74%. For modes i and ii, significance of difference from zero when mGluRIs were activated: *p* = 0.003 and *p* = 0.004, respectively (*n* = 6). However, Homer1a overexpression prevented such a potentiation: mode iii, 3.68 ± 2.01% versus 1.87 ± 2.54%; mode iv, 1.54 ± 1.94% vs −0.02 ± 1.66% (*p* > 0.05 for both cases, *n* = 6; Fig. 6*C*). The same experiment on CGCs (Fig. 7): mode i, 9.88 ± 1.5% versus 3.2 ± 2.18%; mode ii, 9.57 ± 1.93% versus 0.97 ± 2.78%; mode iii, 2.15 ± 2.49% versus 1.76 ± 1.77%; mode iv, 1.97 ± 2.15% versus 2.3 ± 2.12%. Significance of difference from zero when mGluRIs were activated in intact cells: modes i and ii, *p* = 0.006 and *p* = 0.008, respectively (*n* = 6 for both cases, paired Student's *t* test; Fig. 7*C*). In all recordings, the maximum potentiation effect of mGluRIs developed within the 5 ms time window after the application of ligands (Figs. 6, 7).

### mGluRIs modulate NMDAR-generated action potentials (APs) via both Homer- and G-protein signaling pathway

**Figure 8.**
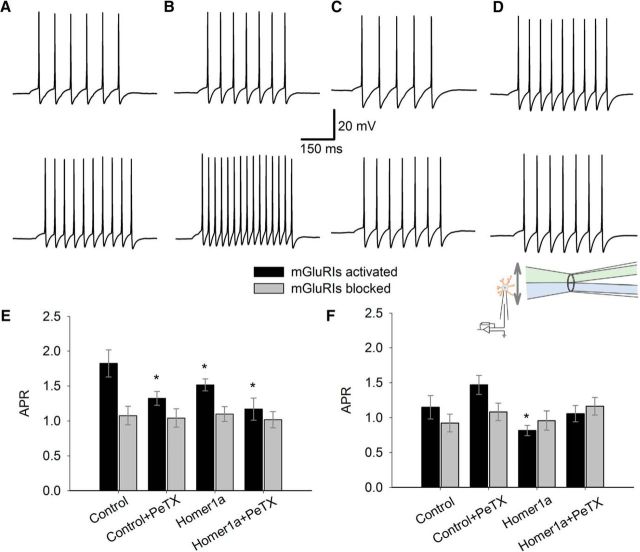
G-protein- and Homer-mediated signaling chains control AP generation via NMDARs. ***A–D***, Example recordings where AP ratio was shifted significantly from values generated by NMDA+Gly. Top, APs evoked by 300 ms application of NMDA 50 μm + Gly 50 μm. Bottom, APs evoked by application of NMDA 50 μm + Gly 50 μm + DHPG 100 μm. ***A***, Intact DGC, PeTX 1 μg/ml in internal solution. ***B***, Homer1a-overexpressing DGC. ***C***, Homer1a-overexpressing DGC, PeTX in internal solution. ***D***, Homer1a-overexpressing CGC. Scale bars apply to ***A–D***. ***E***, Statistical summary for DGCs. Ratios of AP number generated by DHPG+NMDA+Gly to that generated by NMDA+Gly. ***F***, Same as in ***E***, but for CGCs. Color codes apply to ***E*** and ***F***. Inset, Illustration of solution application system (schematic) with θ-glass pipette, which applies solution with mGluRI and NMDAR ligands (“top” channel) at a patched neural cell. During replacement NMDA+Gly → NMDA+Gly+DHPG in “top” channel, patched cell was exposed to perfusion solution flowing from “bottom” channel. Significant difference from control (no PeTX added, no Homer1a overexpression): *p < 0.05; Student's *t* test. *n* = 6–8.

Next, we asked whether and to what extent Homer- and G-protein-mediated mGluRI-NMDAR signaling pathways regulate the generation of neuronal APs. To clarify this, we performed whole-cell recordings from neurons in dispersed cell culture, where APs were evoked by consequent 300 ms applications of the same set of solutions, as in the experiments on membrane patches (Fig. 8). To quantify mGluRI input into AP generation, we used action potential ratio (APR) obtained as a number of APs evoked when NMDARs and mGluRIs are activated with DHPG+NMDA+Gly or mGluRIs are suppressed with NMDA+Gly+FTIDC+FB, divided by number of APs generated in the same cell by NMDA+Gly only.

Here we found that in DGCs blocking of each signaling pathway leads to a significant reduction of APR compared with control value: mode i, control, 1.82 ± 0.19 when mGluRIs were activated versus 1.08 ± 0.14 when mGluRIs were blocked; mode ii, 1.32 ± 0.1 versus 1.04 ± 0.13; mode iii, 1.52 ± 0.09 versus 1.1 ± 0.11; mode iv, 1.17 ± 0.18 versus 1.02 ± 0.12. Significance of difference from control when mGluRIs were activated in modes ii–iv: *p* = 0.044, *p* = 0.047, and *p* = 0.032, respectively (*n* = 6 for all three comparisons, paired Student's *t* test). When Homer- and G-protein pathways were blocked simultaneously in mode iv, the firing frequency observed with DHPG and FTIDC+FB in the applied solution became indistinguishable from unity (i.e., from that generated by NMDA+Gly only): *p* = 0.144 and *p* = 0.426 for DHPG and FTIDC+FB, respectively (*n* = 6, paired Student's *t* test for both APR comparisons; Fig. 8*E*).

In contrast, in CGCs, only Homer1a overexpression triggered significant mGluRI-mediated modulatory effects on APR compared with control: mode i, control, 1.15 ± 0.17 when mGluRIs were activated versus 0.92 ± 0.13 when mGluRIs were blocked; mode ii, 1.47 ± 0.14 versus 1.08 ± 0.12; mode iii, 0.81 ± 0.08 versus 0.95 ± 0.14; mode iv, 1.05 ± 0.11 versus 1.16 ± 0.14. APR in mode iii was significantly lower compared with control and to unity: *p* = 0.042 and *p* = 0.021, respectively (*n* = 8 for both comparisons, paired Student's *t* test). As in DGCs, under mode iv, APR generated with DHPG and FTIDC+FB was indistinguishable from unity: *p* = 0.35 and *p* = 0.08, respectively (*n* = 6, paired Student's *t* test for both comparisons; Fig. 8*F*).

### mGluRI-NMDAR crosstalk modulates synaptic plasticity

**Figure 9.**
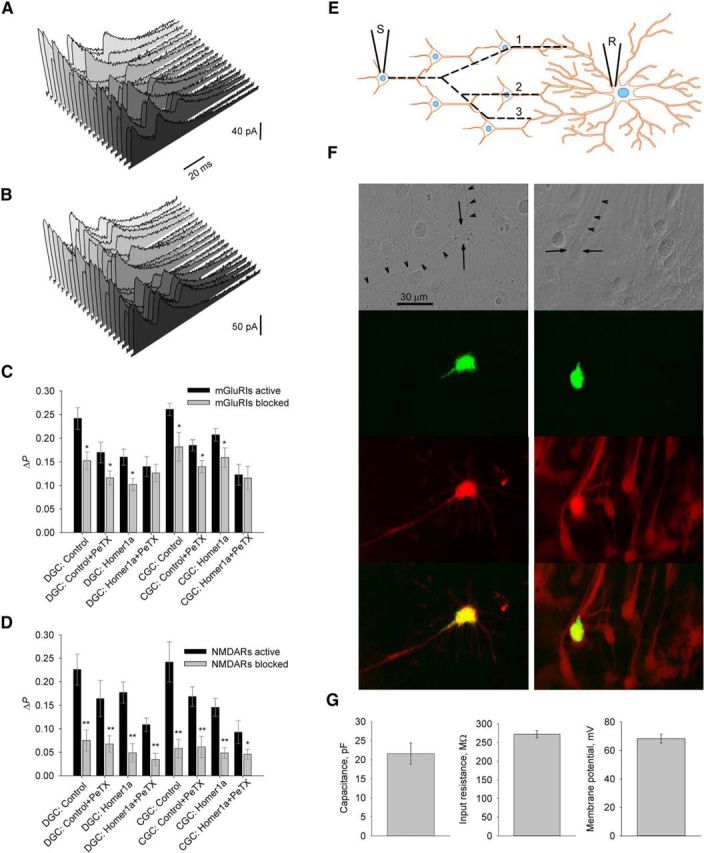
mGluRI-NMDAR crosstalk and synaptic plasticity in cultured hippocampal neurons. ***A***, ***B***, Twenty consecutive EPSCs (inward currents are shown upward) recorded from a cultured neuron in response to stimulation of a nearby neuron before (***A***) and after repetitive paired-pulse stimulation (***B***). There is an increase of second and third EPSC component numbers after paired-pulse stimulation. Scale bars: ***A***, ***B***, 20 ms. ***C***, ***D***, Statistical summary of pathway remodeling induced by paired-pulse stimulation with modulatory impact of mGluRIs (***C***) and NMDARs (***D***). Asterisks above “mGluRIs blocked” and “NMDARs blocked” bars indicate significance of difference between responses obtained with blocked mGluRIs or NMDARs, and when both receptor species are active, under the same experimental mode: *p < 0.05; **p < 0.01; Student's paired *t* test. ***E***, Three hypothetical polysynaptic pathways (dashed lines 1–3) leading from stimulated neuron (S) to the recorded neuron (R) with different transmission delays corresponding to the onset latencies of the three distinct EPSC components. ***F***, Image series illustrating selection of DGCs for further experimental work. Left column, Dispersed culture, solitary DGC with developed morphology (for details, see Materials and Methods). Right column, Dense culture, group of differentiated DGCs. Top to bottom, Images under infra-red DIC; GFP fluorescence (λ_em_ 510 nm) after whole-cell patching; Cy5 fluorescence (λ_em_ 670 nm) after immunostaining for calbindin-D_28k_; overlap of GFP and Cy5 fluorescence patterns. Arrows indicate cell soma. Arrowheads indicate main neurite(s). Scale bar: both columns. ***G***, Statistical summary on electrophysiological properties of DGCs recorded in ***F***; *n* = 6.

As the next step, we set out to clarify the role of two types of mGluRI-NMDAR crosstalk in synaptic transmission and induction of synaptic plasticity. To comprehend this, we used networks of cultured neurons where two polysynaptically connected cells were patched simultaneously. Current injection into one cell (held in current-clamp mode) generated AP with the subsequent poly-component EPSCs recorded from another cell (held in voltage-clamp mode). We interpreted each EPSC component as a signal through separate transmission pathway with specific delay. Under the low-frequency stimulation (1 current injection per 15 s), the probability of occurrence (*P*) remained stable for each EPSC component observed in recorded cell. We thus used the EPSC profile as a tool for quantitative measurement of synaptic efficacy (for more details, see Materials and Methods). To examine synaptic plasticity in recorded network, we applied a train of 50 paired-pulse stimuli with 50 ms interpulse interval at 1 Hz. After that, we monitored changes in *P* (decrease or increase, i.e., Δ*P*) of preexisting EPSC components, which reflect remodeling of signaling pathways (Fig. 9*A*,*B*). Recorded neurons held in modes i–iv allowed assessment of the input of two types of mGluRI-NMDAR crosstalk into modulation of synaptic plasticity.

We found, in DGCs, that blocking of each mGluRI-NMDAR crosstalk pathway induces a significant lowering of Δ*P*: mode i, control: 0.24 ± 0.023 when mGluRIs were activated versus 0.15 ± 0.019 when mGluRIs were blocked; mode ii: 0.17 ± 0.022 versus 0.12 ± 0.015; mode iii: 0.16 ± 0.017 versus 0.1 ± 0.013; mode iv: 0.14 ± 0.021 versus 0.13 ± 0.018. Significance of difference from control for modes ii–iv: *p* = 0.038, *p* = 0.022, and *p* = 0.034, respectively (*n* = 7, Student's *t* test). In modes i—iii, block of mGluRIs caused significant reduction of Δ*P* compared with readout when mGluRIs were active in the same mode: *p* = 0.021, *p* = 0.031, and *p* = 0.036, respectively (*n* = 7, Student's *t* test for all three comparisons; Fig. 9*C*).

In turn, in CGCs blocking of Homer pathway (mode iii) did not trigger significant change of Δ*P* compared with control, unlike the block of G-proteins chains (mode ii) and of both signaling pathways (mode iv): mode i, control: 0.26 ± 0.013 when mGluRIs were activated versus 0.18 ± 0.03 when mGluRIs were blocked; mode ii: 0.19 ± 0.011 versus 0.14 ± 0.013; mode iii: 0.21 ± 0.013 versus 0.16 ± 0.02; mode iv: 0.12 ± 0.022 versus 0.12 ± 0.02. For comparisons of modes ii and iv: to control when mGluRIs activated (*p* = 0.0102 and 0.011, respectively, *n* = 8, Student's *t* test). As in DGCs, in modes i–iii, block of mGluRIs caused significant reduction of Δ*P* compared with readout when mGluRIs were active: *p* = 0.018, *p* = 0.045, and *p* = 0.019, respectively (*n* = 8, Student's *t* test for all three comparisons; Fig. 9*C*).

For both DGCs and CGCs, we did not observe significant difference of Δ*P* values obtained when mGluRIs were blocked under any experimental mode (i–iv), from Δ*P* obtained when mGluRIs were activated, but both mGluRI-NMDAR signaling pathways were blocked (iv): *p* > 0.05 for all comparisons, *n* = 7 (DGCs) and *n* = 8 (CGCs), Student's *t* test (Fig. 9*C*).

As a general rule, alterations of postsynaptic current profile displayed changes in *P* of particular components, but without substantial variation of their amplitude. In DGCs under mode i, amplitude of the first peak after the stimulation train was changed by 17 ± 22% compared with pretrain value; under mode ii, by −11 ± 19%; under mode iii, by +21 ± 18%; under mode iv, by 7 ± 16% (*p* > 0.3 for all comparisons to pretrain value, *n* = 7, Student's *t* test). In CGCs under mode i, first peak amplitude was changed by 14 ± 9%; under mode ii, by −8 ± 13%; under mode iii, by −4 ± 11%; under mode iv, by 9 ± 8% (*p* > 0.2 for all comparisons to pretrain values, *n* = 8, Student's *t* test).

Next, to quantify the role of NMDARs in synaptic plasticity and signaling pathway remodeling, we repeated the same experimental protocol, but with pharmacological block of NMDARs (with 50 μm APV) rather than of mGluRIs. We found that pharmacological silencing of NMDARs induces a significant decrease of Δ*P* values (i.e., much fewer changes of EPSC profiles were observed under all experimental modes; Fig. 9*D*). For DGCs, mode i: 0.075 ± 0.023 when NMDARs were blocked versus 0.23 ± 0.033 when NMDARs were active; mode ii: 0.067 ± 0.018 versus 0.16 ± 0.039; mode iii: 0.049 ± 0.02 versus 0.18 ± 0.022; mode iv: 0.035 ± 0.014 versus 0.11 ± 0.014. For comparisons “APV-active NMDARs”: *p* = 0.0032, *p* = 0.0067, *p* = 0.0054, and *p* = 0.0086 for modes i–iv, respectively (*n* = 7, Student's *t* test). For CGCs, mode i: 0.058 ± 0.02 when NMDARs were blocked versus 0.24 ± 0.42 when NMDARs were active; mode ii: 0.61 ± 0.022 versus 0.17 ± 0.021; mode iii: 0.048 ± 0.011 versus 0.15 ± 0.019; mode iv: 0.046 ± 0.011 versus 0.09 ± 0.024. For comparisons “APV- active NMDARs”: *p* = 0.0028, *p* = 0.0073, *p* = 0.0062, and *p* = 0.023 for modes i–iv, respectively (*n* = 7, Student's paired *t* test).

Finally, as an additional control, we tested accuracy of our cell selection algorithm on DGCs (Fig. 9*F*,*G*). To do this, we patched cultured cells, preselected on visual criteria, with pipette solution containing GFP. If cell electrophysiological parameters were in predefined intervals (membrane capacitance 15–25 pF, membrane potential −65 to 75 mV, input resistance 150–300 mΩ), we performed an accurate withdrawal of patch pipette and captured an image of GFP fluorescence. Afterward, the coverslip with cell culture underwent immunostaining with Cy5 fluorescent dye for the protein calbindin-D_28k_, which is a characteristic DGC marker (Müller et al., 2005) (for more details, see Materials and Methods).

All six cells, which reproduced predefined electrophysiological parameters, have also demonstrated Cy5 fluorescence, thus confirming that chosen selection algorithm circumscribes at least a part of the DGC population. Electrophysiological readouts in this experiment were as follows: membrane capacitance, 21.61 ± 2.77 pF; membrane potential, 68.25 ± 3.12 mV; input resistance, 271.92 ± 9.33 mΩ.

## Discussion

**Figure 10.**
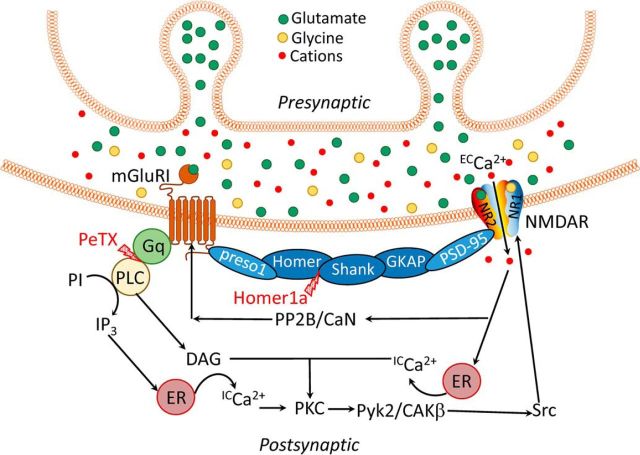
Schematic of mGluRI-NMDAR signaling chains: Homer-mediated (white element titles) and G-protein-mediated (black element titles). Red lightning bolts indicate specific points where G-protein- and Homer-mediated chain were interrupted by PeTX and Homer1a, respectively. mGluRIs and NMDARs are physically connected by scaffolding proteins of postsynaptic density, which transmit modulatory signal through scaffolding proteins in the following order: mGluRI → preso1 → Homer → SH_3_ and multiple ankyrin repeat domains (Shank) → guanylate-kinase-associated protein (GKAP) → postsynaptic density 95 (PSD-95) → NMDAR. Glutamate-containing vesicles of presynaptic bouton, being exocytosed, release glutamate into synaptic cleft. Glutamate binding to mGluRI and glutamate + glycine binding to NMDAR activate both receptors. Phosphorylated NMDAR allows influx of extracellular calcium (^EC^Ca^2+^) into cell; this triggers release of intracellular calcium (^IC^Ca^2+^) from ER. Activation (phosphorylation) of mGluRI, induced by glutamate binding, triggers activation of the G-protein G_q_, subsequently activating phospholipase C (PLC). PLC initiates conversion of phosphoinositide (PI) to inositol 1,4,5-triphosphate (IP_3_) and subsequent activation of diacylglycerol (DAG); IP_3_ initiates release of intracellular Ca^2+^. DAG, being combined with increased Ca^2+^ concentration due to NMDAR activation, causes phosphorylation of protein kinase C (PKC). PKC initiates phosphorylation of proline-rich tyrosine kinase/cell adhesion kinase β (Pyk2/CAKβ), and subsequently the cellular tyrosine kinase protein (Src). In turn, SRC potentiates NMDAR. Negative feedback loop occurs after Ca^2+^ influxed through NMDARs activates Ca^2+^-dependent protein phosphatase 2B/calcineurin (PP2B/CaN), which dephosphorylates (i.e., at least partially deactivates) mGluRIs (Matosin and Newell, 2013).

In this work, we examined the mGluRI-NMDAR crosstalk through two pathways: G-protein-mediated and Homer-mediated (Fig. 10). At a short time interval (units of milliseconds), the only significant result of such a crosstalk, similar for DGCs and CGCs, was a facilitation of NMDAR response triggered by mGluRIs and delivered through Homer-containing scaffold. On a long time interval (from hundreds of milliseconds to seconds), this facilitation overlaps with slower modulatory impact delivered through G-protein-initiated signaling cascade. The type of G-protein-delivered impact (facilitation or depression) depends on the particular cell type. The end result of mGluRI-NMDAR crosstalk is therefore cell-specific and depends on a registration timescale.

In DGCs, the mGluRIs generated a significant potentiation of long, equilibrated NMDAR response under control conditions (Fig. 1) in line with previous reports (Awad et al., 2000). The potentiation generated by mGluRIs in Homer1a-overexpressing DGCs (i.e., delivered through G-protein pathway only) was significantly higher than that delivered via both G-protein and Homer pathways in intact cells (Fig. 1*C*). This implies the competitive nature of the two, albeit unidirectional, signals. In contrast, in CGCs, only the G-protein pathway, being activated alone in Homer1a-overexpressing cells, induced a significant downregulation of NMDAR effect, thus resembling previous observations (Bertaso et al., 2010). No significant effect of any direction was generated by the Homer pathway only or when both pathways were active (Fig. 2). The plausible explanation is that the readout of the Homer-delivered effect on equilibrated response in CGCs is below the applied method's sensitivity, but this pathway functionally prevails over G-protein signaling and thus muffles the downregulation delivered through G-proteins.

For both DGCs and CGCs, no significant effect of mGluRIs was demonstrated when Homer- and G-protein-controlled pathways were simultaneously blocked (Figs. 1*C*, 2*C*), which implies the lack of significant input from other mechanisms of mGluRI-NMDAR crosstalk. Next, we observed the effect of DHPG in nucleated patches (Figs. 1, 2, 4–7) and in whole-cell (Figs. 8, 9), but not in outside-out patches (Fig. 3). This proves the critical dependence of mGluRI-NMDAR interaction from cytoplasmic elements and/or elements not anchored into outer cell membrane. On top of that, the lack of DHPG effect in outside-out patches proves that DHPG does not activate NMDARs directly, acting as NMDA coagonist (Contractor et al., 1998).

An alternative pathway of mGluRI-NMDAR crosstalk is modulation of assembly of Homer-containing protein scaffold, where repetitive NMDAR activation shifts equilibrium to disassembled state (Moutin et al., 2012) (Fig. 10). Can this pathway be involved into modulation of effects studied in our research? Moutin et al. (2012) demonstrated localization of such a process exclusively in postsynaptic compartments; thus, it could exert an effect in whole-cell experiment (Fig. 9). In contrast, axo-somatic synapses were reported for DGCs (Toni et al., 2007); however, to the best of our knowledge, not for CGCs. Therefore, similar results of Homer-delivered rapid effects obtained after repetitive NMDAR activation on NPs pulled from both DGCs and CGCs (Figs. 6, 7) argue against the presence of NMDAR-induced disruption of Homer-containing interlink at least in this type of experiment.

In contrast to the equilibrated NMDAR response (Figs. 1, 2), where the effect of mGluRIs was specific to the cell type under study (potentiation of NMDAR response in DGCs, suppression in CGCs), at a short time interval after rapid ligand application, the mGluRIs potentiate an NMDAR response amplitude in both cell types (Figs. 6, 7), resembling short-term mGluRI effects, which have been documented previously (Kinney and Slater, 1993; Rossi et al., 1996; Sylantyev et al., 2013). This potentiation is insensitive to PeTX but does not develop in Homer1a-overexpressing cells, thus suggesting the Homer protein chain as the only underlying mechanism.

Despite the apparent modulatory impact of mGluRI-NMDAR crosstalk on neural signaling revealed in our experiments, it is still an important question: whether our data represent a typical set of effects. Or this is a result of specific combination of experimental conditions, such as the age of tested cells and extrasynaptic, rather than synaptic, localization of recorded receptors in experiments on membrane patches? It was reported earlier that synaptic and extrasynaptic pools of NMDARs display different proportions of NR1 and NR2 subunits (Barria, 2007), whereas different receptor subunits interact differently with PSD-95 protein (Bassand et al., 1999; Al-Hallaq et al., 2007), which links them to mGluRI via the Homer-containing scaffold (Fig. 10). On top of that, expression levels of different NMDAR subunits (Monyer et al., 1994) and different types of Homer protein (Shiraishi et al., 2004) are age-dependent. However, it was shown that NMDARs and Homer proteins make functional clusters throughout all developmental stages in both extrasynaptic and synaptic loci of cultured neurons (Shiraishi et al., 2003). Apart from that, studies on cultured neurons of different age demonstrated effects of mGluRI-Homer-NMDAR interaction similar to our present observations. These effects were found in CGC NPs (Sylantyev et al., 2013) (i.e., were generated by extrasynaptic receptors) and in a whole-cell (Bertaso et al., 2010) (i.e., were generated primarily by synaptic receptors). Similarly, G-protein-mediated mGluRI-NMDAR modulation was demonstrated in cultured neurons from the earliest stages of their development (Hilton et al., 2006) to maturation (Lea et al., 2002); such a modulation occurs both in synapses (Kwon and Castillo, 2008) and at extrasynaptic membrane (our data). These observations suggest that mGluRI-NMDAR signaling pathways researched in our study preserve their functionality independently from particular receptors localization and the cell age. However, their relative input into integrated modulatory tone may vary depending on a stage of the neuron development.

NMDARs are widely recognized to play a pivotal role in long-term synaptic plasticity in the CNS (Malenka and Nicoll, 1993; Paoletti et al., 2013). In our work, we demonstrated two mGluRI-NMDAR crosstalk pathways to be modulators of Δ*P* (i.e., of NMDAR input into changes of synaptic strength after series of paired stimuli) (Fig. 9*D*). Therefore, we found that G-protein-controlled and Homer-controlled signaling pathways between mGluRI and NMDAR play a significant role in synaptic plasticity and efficacy.

Our further experiment on the signaling pathway remodeling demonstrated that NMDAR silencing with APV reduces significantly variability of neural network synaptic transmission and synaptic strength (Δ*P*), which is consistent with earlier observations of involvement of synaptic NMDARs into long-term plasticity (Bi and Poo, 1999; O'Riordan et al., 2018). However, even when both mGluRI-NMDAR signaling pathways were blocked (mode iv) and APV added, we did observe a certain degree of Δ*P* (Fig. 9*D*,*E*). This may reflect involvement of mGluRI- and NMDAR-independent mechanisms, such as modification of GABA-ergic conductance (Linden and Connor, 1995) and/or activity-dependent modifications of neuronal excitability (Turrigiano et al., 1994).

Our experiments on neural networks demonstrated that similar pattern of stimuli can induce opposite changes along different signaling pathways (increase or decrease of *p*). Modulation of EPSC profiles without significant impact on response amplitudes suggests that the chosen pattern of stimulation had caused variation of synaptic strength at remote synaptic connections, thus changing the probability of a signal transmission by different pathways connecting stimulated and recorded cell, with variable interplay between these pathways. This type of modification is consistent with a paradigm of distributed storage and representation of information in neural networks (Churchland and Sejnowski, 1992; Bliss and Collingridge, 1993).

Constitutive agonist-independent activity of G-protein-coupled receptors was repeatedly detected in various experimental setups and may substantially impact cell functioning and intercellular signaling (Milligan, 2003). However, the absence of any detectable difference between mGluRI effects under control and when mGluRI inverse agonists FTIDC and FB are applied in all types of experiments suggests that NMDAR effects triggered by NMDA+Gly alone (i.e., without activation of mGluRIs) are not modulated by spontaneous mGluRI activity. Thus, we presume that the nonsignificant effects of mGluRI activation in intact CGCs (Fig. 8*F*) is due to the overlap of Homer- and G-protein-delivered signals of opposite sign, rather than the result of, for instance, the majority of mGluRIs in the active state (due to spontaneous activation) before application of DHPG.

Therefore, in our study, we found the functional mechanism of mGluRI-NMDAR interaction in DGCs and CGCs to be as follows (Fig. 10): mGluRIs, when activated, modulate NMDARs simultaneously through Homer- and G-protein-controlled pathways via changes of NMDAR opening probability. The Homer-containing protein interlink delivers potentiating signal to NMDARs in both cell types, whereas the G-protein-mediated signal is cell-specific: it potentiates NMDAR function in DGCs and suppresses it in CGCs. Fast Homer-transduced effect is more prominent at short time intervals after mGluRI activation, whereas slower developing G-protein-delivered effect has an advantage on long-term intervals. Additionally, Homer-mediated potentiation tone prevails over modulation delivered by G-protein cascade, although it causes smaller absolute effect.

The hippocampus has been recognized as a key brain area in schizophrenia development (Harrison, 2004), associated with decreased NMDAR function (Gao et al., 2000). Here we observed an increase of NMDAR conductivity in Homer1a-overexpressing DGCs (Fig. 1*C*) due to higher mGluRI-triggered potentiation delivered through a G-protein chain, rather than through a competing Homer interlink (Figs. 1*C*, 4*C*, 8*E*). This suggests a plausible mechanistic explanation for haloperidol and clozapine's anti-schizophrenia effects associated with the upregulation of Homer1a synthesis (Polese et al., 2002).
